# Effect of Wort Boiling System and Hopping Regime on Wort and Beer Stale-Flavor Aldehydes

**DOI:** 10.3390/foods12163111

**Published:** 2023-08-18

**Authors:** Alexandr Mikyška, Karel Štěrba

**Affiliations:** 1 Research Institute of Brewing and Malting, Lípová 511/15, 120 00 Prague, Czech Republic; info@beerresearch.cz; 2 Faculty of Agrobiology, Food and Natural Resources, Czech University of Life Sciences Prague, Kamýcká 129, 165 00 Prague, Czech Republic

**Keywords:** carbonyls, Strecker aldehydes, pressurized wort boiling, hopping regimes, wort, beer

## Abstract

The main factor responsible for the sensory aging of beer is the increase in off-flavor aldehydes during beer storage. In pilot brews (200 L) of pale lager beer with different hopping regimes and wort boiling systems, 15 carbonyls were monitored using the GC-MS method. Factor analysis revealed several groups of aldehydes with similar behavior during wort boiling. The concentration of most of them decreased with atmospheric wort boiling and increased with the time and energy-saving pressurized boiling system. Wort clarification was a critical step because of the increase in carbonyl concentration, with the level of most carbonyls being higher in the final wort compared to sweet wort. The hopping regimes only affected the level of 3-methylbutan-2-one in the wort. The concentration of carbonyls decreased significantly (30–90%) during fermentation, except for trans-2-butenal, which increased by 59% on average, likely due to the release from imine complex. The concentration of free aldehydes in the fresh beers was similar for all variants used, but the pressurized wort boiling system could result in lower sensory stability of the beer due to the release of aldehydes from inactive complexes formed during fermentation. This aspect requires further investigation.

## 1. Introduction

Beer is one of the most popular beverages in the world, with an annual production of 1800 million hectoliters. Unfortunately, due to its complex composition, it is subject to significant flavor changes during storage. Despite decades of extensive research, beer flavor instability remains a challenge for both brewing and malting industries [1]. Carbonyl compounds are considered to be the main cause of sensory beer aging, the most frequently mentioned being Strecker aldehydes (2-methylpropanal, 2-methylbutanal, 3-methylbutanal, methional, benzaldehyde, phenylacetaldehyde), 2-furfural and lipid-degradation products (hexanal, trans-2-nonenal) [1,2,3,4]. During beer storage, carbonyl compounds can be formed de novo or released from existing complexes with sulfites or cysteine [5,6]. Approximately 85% of the Strecker aldehydes found in aged beer originate from the brewhouse [7,8]. Therefore, the raw material–technological aspects of the formation and changes in carbonyl compounds are already paid attention during the beer production process, as they can be of great importance for the quality of fresh beer and its sensory stability [1,2,3,7,9].

Carbonyl compounds in wort partly originate from malt and are partly formed during sweet wort production [1,3]. The concentration of aldehydes is reduced by boiling the wort to the equilibrium level between evaporation and the formation of new compounds; precursors of Strecker aldehydes in the wort are amino acids and Maillard reaction intermediates, and lipid degradation with the formation of linear or unsaturated aldehydes also occurs [3]. An exception is 2-furfural, the concentration of which increases during wort boiling and is therefore considered to be an indicator of heat load [10]. The addition of hops can improve the antioxidant status of the wort and suppress the formation of some carbonyls [7]. Physical factors influencing the level of carbonyls during wort boiling are evaporation and temperature [3]. Different wort boiling systems result in different carbonyl concentrations in the wort [11].

Clarification and cooling of the wort is another stage where, depending on specific conditions, the concentration of carbonyls can increase [12]. The fermentation process significantly reduces the level of aldehydes, likely due to the reductive metabolism of yeast [13,14] and the production of sulfite, which can covalently bond the carbonyls [3,15].

The findings published to date on the influence of the wort boiling process on the content of stale-flavor carbonyl compounds in wort and beer, apart from the issue of hopping, are part of works devoted to the investigation of the influence of malt, mashing or the whole hot section of beer production. The process parameters of wort boiling have not been independently tested. In particularly, the effect of advanced energy and time-saving pressurized wort boiling systems on the stale-flavor carbonyls in wort and beer has not yet been sufficiently investigated. The aim of our work was therefore to investigate, under the same experimental conditions, the influence of the (i) boiling time, (ii) wort extract concentration, (iii) hopping regime and (iiii) pressurized boiling system on the dynamics of carbonyl formation/evaporation during boiling with respect to their concentration in the final wort and subsequently in the beer.

## 2. Materials and Methods

### 2.1. Preparation of Wort and Beer

Pilot brews (200 L) of a pale lager with one replication were carried out in the multifunctional pilot brewhouse (Kaspar Schulz, Germany). All-malt wort was produced from Pilsner malt using a single decoction process. The brews were lautered to an equal final wort volume of 200 L. The retention time in the lauter tun was 20 min before lautering. The wort extract was 11%, except for wort 2 (15%).

Atmospheric wort boiling (ATM) and two low-overpressure systems (pressure boiling (PWB: overpressure, 150 mbar; temperature, 102 °C) and dynamic pressure boiling (PDWB: overpressure maximum, 150 mbar; temperature 102 °C; three pressure peaks; identical increase and decrease in overpressure to atmospheric pressure) were tested. Pellets 90 of Saaz (PE: α-acids, 3.35%), CO_2_ hop extract of Magnum (EX: α-acids, 49.4%) and a combination of both in the ratio 1:1 (EX + PE) were used. An overview of the experimental variants is shown in Table 1. The selected variants of brewing experiments aimed to elucidate the influence and significance of the effect of wort boiling time, wort concentration, hop raw material in atmospheric wort boiling systems and the influence of time- and energy-saving pressurized wort boiling systems, with a focus on the dynamics of carbonyl formation/evaporation during wort boiling and its changes in subsequent brewing operations. The hop raw materials were dosed in relation to the target bitterness of the beer of 30 mg/L iso-α-acids. A higher dose was therefore used for ATM2 (beer dilution with water) and ATM3 (reduced boiling time, lower α-acid isomerization). The residence time in the whirlpool was 20 min, then the wort was cooled down to a fermentation temperature of 10 °C and aerated to a dissolved oxygen concentration of 7.5 mg/L.

Primary fermentation with lager yeast strain RIBM 95 took place in cylindrical conical tanks at a maximum temperature of 12 °C, followed by three-week maturation at a temperature of 1–2 °C. The beer was then filtered, bottled and pasteurized. The wort samples were taken as follows: sweet wort at the onset of the boil (SW), after 30 min of wort boiling (W30), after 60 min of wort boiling (W60), hot final wort at the end of the boil (HW) and clarified and cooled final wort (CW). Wort 3 after 60 min of wort boiling was labelled as hot final wort.

### 2.2. Chemical Analysis

The analyses of wort extract, original gravity and alcohol content in beer were carried out on the DMA 4500 M density meter, coupled with Alcolyzer ME (Anton Paar GmbH, Graz, Austria). Wort samples were filtered before analysis. Beer samples were filtered and degassed before analysis.

The bitterness of wort and beer was determined according to Analytica EBC, methods 8.8 and 9.8 [16]. A total of 10 mL of wort or beer was placed in an Erlenmeyer flask, and 0.5 mL of 6 M HCl and 20 mL of isooctane were added. After sealing, the flask was shaken vigorously in a shaker for 15 min. After shaking, the mixture was centrifuged at 3000 rpm for 3 min. In the isooctane layer, the absorbance was measured immediately at 275 nm against pure isooctane. The bitterness value was calculated according to the following formula: Bitterness = 50 × Absorbance _275 nm_.

The thiobarbituric acid index (TBA) was determined using the MEBAK method [17]. A total of 10 mL of filtered wort or filtered and degassed beer were placed in a test tube, and 5 mL of 0.02 M thiobarbituric acid was added. The mixture was shaken well and placed in a water bath heated to 70 °C. After 70 min, the tube was cooled to 20 °C. The absorbance of the sample was measured at 448 nm against distilled water immediately after cooling. The blank was prepared in the same way, except that 5 mL of 90% acetic acid was added to 10 mL of sample prior to preparation. The thiobarbituric acid index was calculated according to the following formula: TBA = (Absorbance _sample_ − Absorbance _blank_) × 10. Carbonyl compounds were determined by the GC-MS method according to Čejka et al. [18] with minor modifications. The following 15 carbonyl compounds, 13 aldehydes and 2 ketones were quantified: acetaldehyde (ACA), acetone (ACO), 2-methylpropanal (2MP), 3-methylbutan-2-one (3MO), 2-methylbutanal (2MB), 3-methylbutanal (3MB), trans-2-butenal (T2B), hexanal (HEX), heptanal (HEP), octanal (OCT), furfural (FUR), trans-2-octenal (T2O), trans-2-nonenal (T2N), benzaldehyde (BEN) and phenylacetaldehyde (PAA). The standards, the internal standard 3-fluorobenzaldehyde and the derivatization reagent O-(2,3,4,5,6-pentafluorobenzyl) hydroxylamine hydrochloride (PFBOA) were purchased from Merck (Darmstadt, Germany). The wort or beer samples were adjusted to pH 4.4 using 0.1% H_3_PO_4_. Five milliliters of sample were placed into the test tube, and 20 µL of internal standard (concentration 120 mg/L), 50 µL of Na_2_S_2_O_3_.5 H_2_O (0.1 M), and 500 µL of PFBOA were added. The mixture was thoroughly shaken and allowed to stand at room temperature to allow the derivatization reaction to proceed. After 1 h, the reaction was stopped by adding 50 µL of 9 M H_2_SO_4_. The PFBOA derivatives were subsequently extracted with 1 mL of n-hexane. The hexane layer was removed, washed three times with 5 mL of 0.05 M H_2_SO_4_ and anhydrous sodium sulfate was added to remove possible traces of water from the extract. GC-MS analysis was carried out on the Trace GC Ultra gas chromatograph coupled with mass spectrometer DSQ II (Thermo Fisher Scientific, Waltham, MA, USA). The polar column TG-WAX MS (30 m, 0.25 mm i.d., 0.25 µm film thickness) was used. The carrier gas flow was set at 1.2 mL/min, 1 µL of hexane extract was injected in a splitless mode, and the splitless time was 1 min. The injection temperature was 250 °C. The oven temperature was held at 60 °C for 2 min and then increased to 240 °C at 6 °C/min. The compounds were determined in SIM mode.

All wort and beer analyses were performed in duplicate. For better comparison, the concentrations of carbonyl compounds and TBA in wort and beer were recalculated to 11% *w*/*w* original gravity. The data are presented in the Supplementary Materials (Tables S1 and S2).

### 2.3. Sensory Evaluation of Wort

Sensory evaluation was carried out by a 12-member panel of trained evaluators. Wort samples were assessed immediately after cooling. The list of obligatory sensory descriptors was palate fullness, bitterness, character of bitterness, sweetness, astringency and sourness. The list of additional descriptors was grainy, malty, biscuity, nutty, honey-like, papery, musty, grassy, caramel, bitter caramel, bready, medicinal, candy floss, mouse, chemical, burnt, roasted, floral, fruity, green grass, herbal, spicy and woody. All other parameters were evaluated in the rank 0–5 (zero: none; one: very weak; 2: weak; 3: average; 4: strong; 5: very strong). An optional parameter was considered as relevant when at least one third of the panel found it present in the sample. The results are shown in Table S3.

### 2.4. Statistical Evaluation

Analytical results were evaluated by the software STATISTICA 12 (Statsoft Inc., Tulsa, OK, USA), version 12.0.1133.15. The results of the analyses of the experimental variants of wort boiling were tested by one-factor analysis of variance, and Tukey’s HSD test at the α < 0.05 level was used to identify differences between variants.

## 3. Results and Discussion

### 3.1. Boiling Time and Hopping

**Figure 1:**
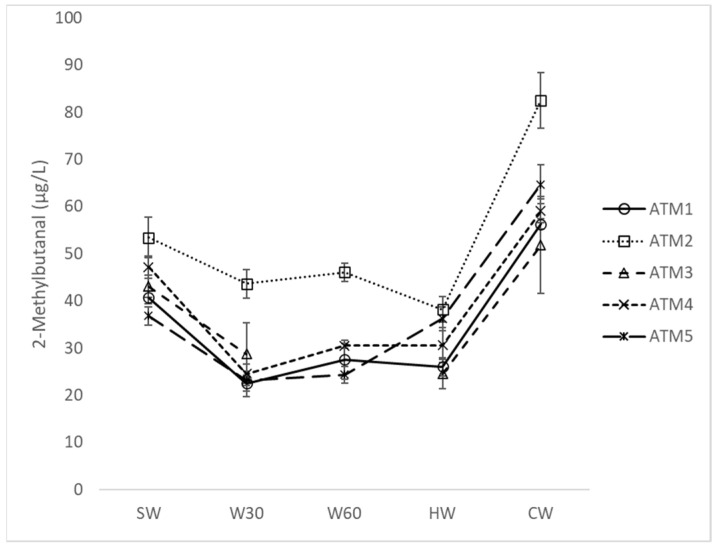
Concentration changes in 2-methylbutanal during atmospheric wort boiling. SW: sweet wort; W30: 30 min of wort boiling; W60: 60 min of wort boiling; HW: hot wort; CW: cold wort.

**Figure 2:**
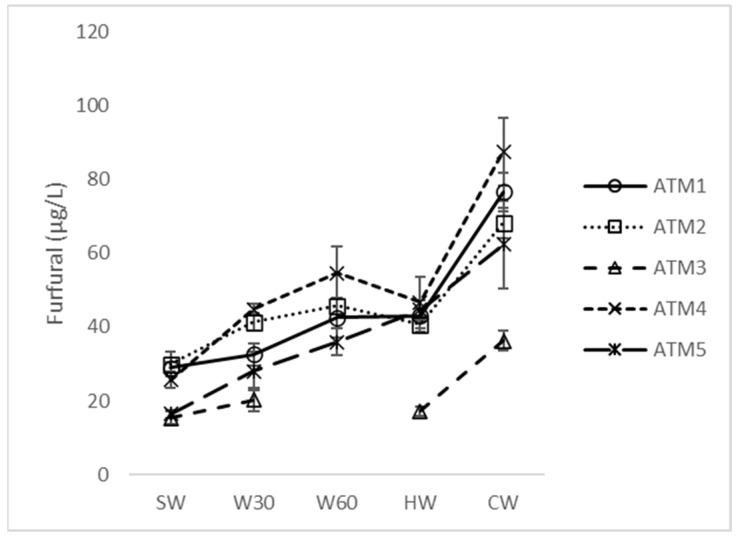
Concentration changes in furfural during atmospheric wort boiling. SW: sweet wort; W30: 30 min of wort boiling; W60: 60 min of wort boiling; HW: hot wort; CW: cold wort.

Comparing five samples prepared by the ATM system (Table 1), a similar trend in changes in concentration was observed for most of the monitored compounds (Table S2). During wort boiling, the concentration of acetaldehyde, acetone, 2-methylpropanal, 2-methylbutanal (Figure 1), 3-methylbutanal, hexanal, heptanal, benzaldehyde and phenylacetaldehyde decreased to a certain level or did not change if the starting concentration was similar to the final level. On the contrary, the concentration of furfural (Figure 2) increased continuously from SW to HW, with the lowest concentration in CW for ATM3 with a shorter boiling time and lower heat load according to TBA (Table 2). The unusual behavior of furfural is due to its significantly lower volatility and the different reaction mechanism of its formation by the Maillard reaction [10].

Some hop constituents are considered to be effective retarders of beer sensory aging by suppressing the formation of stale-flavor aldehydes [7]. Hop α-acids act as free radical scavengers and transition metal chelators [19], and polyphenols show a similar function [19,20]. A higher hop dose (ATM3 vs. ATM1), hop polyphenols (ATM1 vs. ATM4) and a split dose (ATM5 vs. ATM1 and ATM4) did not significantly affect the concentration of the mentioned carbonyls in either cold wort or beer. During wort clarification and cooling, the concentration of 2-methylpropanal, 3-methyl-2-butanone, 2-methylbutanal, 3-methylbutanal, heptanal and, in some samples, acetaldehyde, trans-2-butenal, furfural and phenylacetaldehyde increased, presumably due to a change in the equilibrium between newly formed and evaporated compounds [5,21]. A similar increase in this technological step has been described by Ditrych et al. [12], although the differences observed in this experiment were higher. The highest relative increase in most carbonyls was observed in the high-gravity brew ATM2, suggesting that wort extract concentration has a significant effect on the final carbonyl content. The same conclusion was reached by De Clippelleer et al. [22], who tested the influence of malt origin on the sensory stability of beer, represented by similar aldehyde markers. The rate of change between HW and CW in the content of individual carbonyls was different. Strecker aldehydes 2-methylpropanal, 2-methylbutanal and 3-methylbutanal, as well as 3-methylbutan-2-one, heptanal and furfural showed a significant increase between HW and CW (80–150% on average).

**Figure 3:**
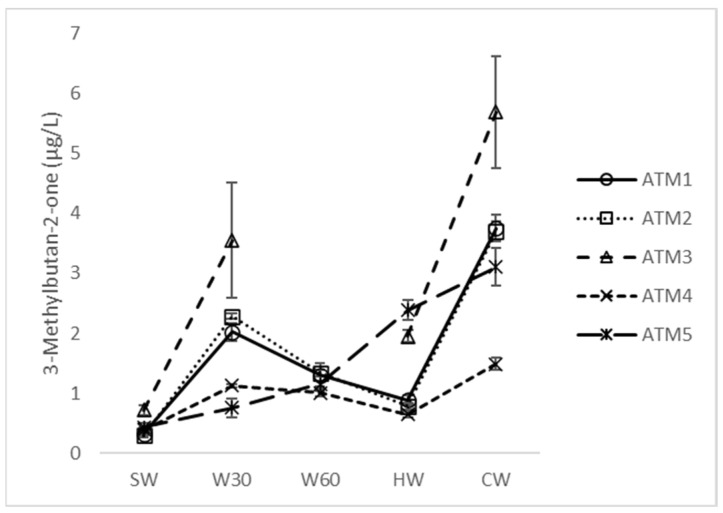
Concentration changes in 3-methylbutan-2-one during atmospheric wort boiling. SW: sweet wort; W30: 30 min of wort boiling; W60: 60 min of wort boiling; HW: hot wort; CW: cold wort.

The split hop dosage resulted in a lower or insignificant increase in the concentration of these aldehydes in the cold wort. An insignificant increase between HW and CW was observed for the Strecker aldehydes benzaldehyde and phenylacetaldehyde, as well as hexanal, trans-2-butenal, trans-2-octenal and trans-2-nonenal. In contrast, Mertens et al. [19] and Wietstock et al. [23] reported suppression of carbonyl formation in a split dose due to the antiradical and chelating ability of hop α-acids. A specific behavior was observed for 3-methylbutan-2-one (Figure 3), which is also considered to be a strong beer-aging indicator [24].

Its concentration increased from a low level in the sweet wort during the first 30 min of boiling and then decreased. ATM5 (split hop dose) showed a constant increase throughout the process, with the highest concentration found in ATM3 (highest hop dose). The formation of 3-methylbutan-2-one from iso-alpha acids generated during boiling from hop α-acids has been proposed by some authors [1]. However, given the lower levels found in ATM4 (hop extract), it is reasonable to assume that other hop constituents, likely polyphenols, also play a role in the 3-methylbutan-2-one formation mechanism. As the concentration of 3-methylbutan-2-one in cold wort is significantly lower than that of Strecker aldehydes, the main source of off-flavor carbonyls in beer are obviously aldehydes or their precursors derived from malt [5,22].

### 3.2. Atmospheric and Pressurized Boiling

**Figure 4:**
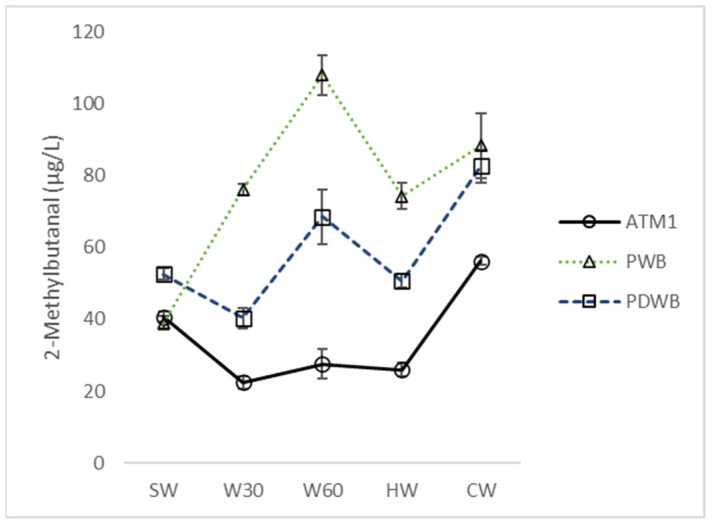
Concentration changes in 2-methylbutanal during wort boiling in different boiling systems. SW: sweet wort; W30: 30 min of wort boiling; W60: 60 min of wort boiling; HW: hot wort; CW: cold wort.

The equilibrium between newly formed and evaporated compounds was significantly altered in the pressurized systems. In the PWB system, the concentration of almost all compounds monitored increased up to 60 min of boiling. After pressure release (HW), the concentration decreased and changes in the whirlpool were inconclusive except for 2-methylbutanal (Table S2, Figure 4), 3-methylbutanal and heptanal. In the PDWB system, the concentration of carbonyls varied according to the pressure changes. Consequently, the changes in carbonyls during trub separation were similar to those in the ATM system. Compared to ATM1 with the same hop dosage, the concentrations of acetaldehyde, acetone, 2-methylpropanal, 3-methylbutan-2-one, 2-methylbutanal (Figure 4), 3-methylbutanal, hexanal, benzaldehyde and phenylacetaldehyde in CW were significantly higher for both pressurized systems. Similar results were obtained by Herrmann et al. [11], who found higher levels of benzaldehyde, phenylacetaldehyde, hexanal, heptanal and furfural in wort from the PDWB system than from the atmospheric system.

### 3.3. Changes of Carbonyls during Wort Boiling

The changes in Strecker aldehydes (2MP, 2M, 3MB, PHE) of fatty acid carbonyls (HEX, T2N) and furfural during atmospheric boiling are consistent with the behavior of these compounds found by Ditrych et al. [12] when tracing carbonyls from malt to cold wort, but the losses in our experiment were much lower. Thus, in contrast to Ditrych et al. [12], we found significantly higher concentrations of these carbonyls in cold wort compared to sweet wort.

**Figure 5:**
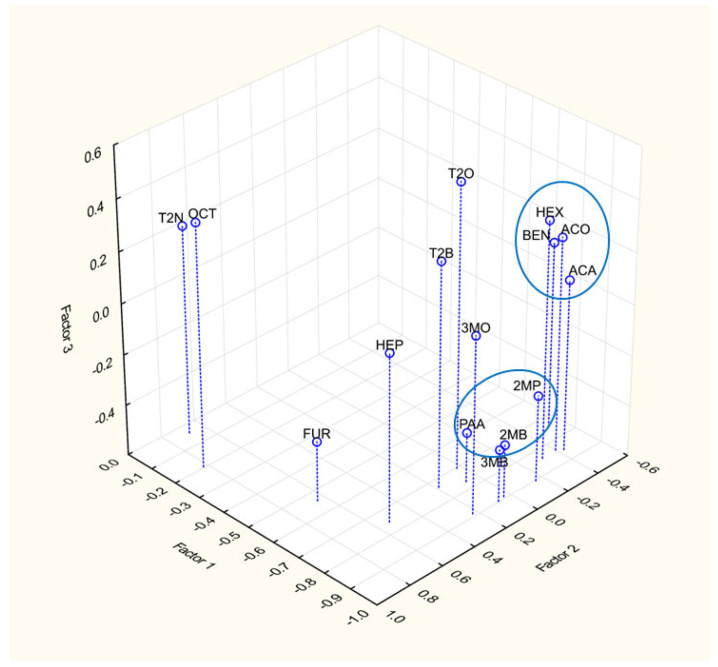
Graphical representation of the factor loadings for the first three factors from the factor analysis. Acetaldehyde (ACA), acetone (ACO), 2-methylpropanal (2MP), 3-methylbutan-2-one (3MO), 2-methylbutanal (2MB), 3-methylbutanal (3MB), trans-2-butenal (T2B), hexanal (HEX), heptanal (HEP), octanal (OCT), furfural (FUR), trans-2-octenal (T2O), trans-2-nonenal (T2N), benzaldehyde (BEN) and phenylacetaldehyde (PAA). Blue circles mark carbonyls with similar behavior.

Some authors have reported similar patterns in the behavior of Strecker aldehydes and fatty acid aldehydes [5,12]. Therefore, we evaluated the concentrations of carbonyls during wort boiling by factor analysis, and a graphical representation of the factor loading for the first three factors (eigenvalues 8.46, 2.69 and 1.40 for the first, second and third factors, respectively) is shown in Figure 5. At the bottom is the group consisting of Strecker aldehydes 2-methylpropanal (2MP), 2-methylbutanal (2MB), 3-methylbutanal (3MB) and phenylacetaldehyde (PHE). On the top right is the group consisting of acetaldehyde (ACA), acetone (ACO), benzaldehyde (BEN) and hexanal (HEX). Other components do not seem to form groups in this arrangement and only octanal (OCT) and trans-2-nonenal (T2N) are projected together, but no trend in changes during the process was observed for either of them. Interestingly, benzaldehyde is grouped with compounds formed by high-temperature exposure of malt compounds but by a different mechanism than the Strecker degradation of amino acids. This suggests that its formation during wort boiling is more influenced by other mechanisms as a possible precursor of benzaldehyde was suggested, i.e., coumaric acid [21]. However, further experiments are needed to confirm and clarify this phenomenon.

**Figure 6:**
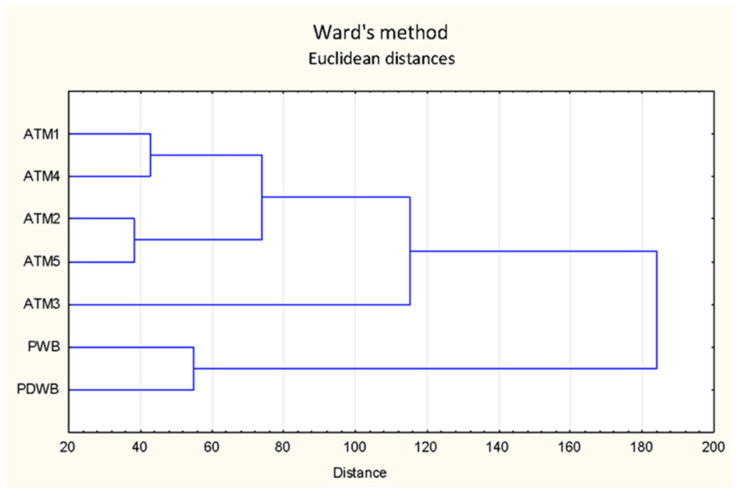
Carbonyls clustering in cold wort.

The overall view of the variability in the concentration profile of carbonyls depending on the wort boiling variant reflects the above-discussed differences in the content of individual substances. Cluster analysis of the set of mean values of individual carbonyls in CW showed a separation of the atmospheric and pressurized systems at the first level of the hierarchy (Figure 6). From the group of atmospheric boils, the variant with a short boiling time (ATM3) was closest to the pressurized system. The variants with hop pellets and hop extract (ATM1 and ATM4) show minor mutual differences.

### 3.4. Beer

**Figure 7:**
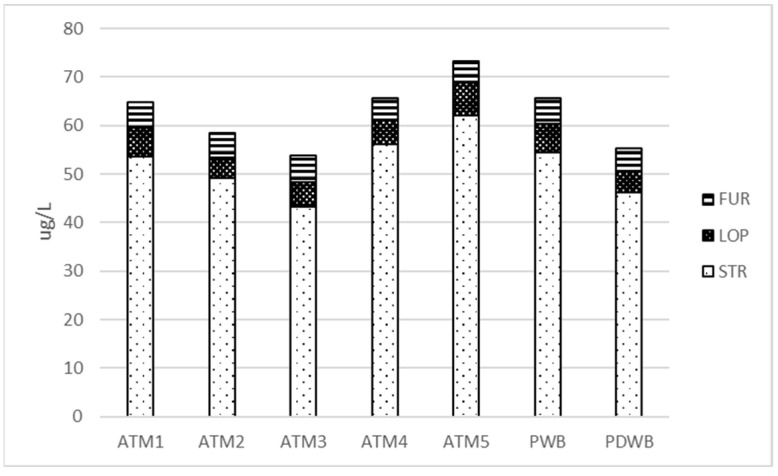
Concentration of carbonyl groups in beer. STR: Strecker degradation aldehydes (2MP, 2MB, 3MB, BEN, PAA); LOP: lipid oxidation products (T2B, HEX, HEP, OCT, T2O, T2N); FUR: furfural.

Significant changes in carbonyl content occurred during fermentation. The reduction in aldehydes in this technological step has been attributed to the reducing capacity of yeast [14,15]. Other pathways include the formation of ‘masked aldehydes’ of bisulfite adducts with sulfur dioxide, complexes with the sulfur-containing amino acid cysteine and the formation of imines with other amino acids [6]. The levels of 2-methylpropanal, 3-methylbutan-2-one, 2-methylbutanal, 3-methylbutanal, hexanal, heptanal, furfural and phenylacetaldehyde decreased significantly in all variants studied, ranging from about 80 to 95% of the CW value. Decreases of 50 to 80% were observed for acetone, trans-2-octenal and trans-2-nonenal. Changes in benzaldehyde and octanal concentrations were variable and relatively small (up to about 50%). The decrease in Strecker aldehydes and hexanal was higher in the overpressure boiling systems, likely due to the higher concentration in CW (see Table S2). Higher decreases were also observed in 2MB and 3MB in the ATM2 variant, likely due to dilution after fermentation. The lowest relative decrease in furfural was observed in ATM3 with a shorter boiling time, and its concentration in the beers was at the same level. The resulting differences in the levels of Strecker aldehydes, lipid oxidation aldehydes and furfural in the beer were different from those in the wort, and the levels in the pressurized systems were comparable to or lower than those in the atmospheric system (Figure 7).

In contrast, acetaldehyde increased due to its production by yeast metabolism as an intermediate of ethanol formation (see Table S2). Higher levels of trans-2-butenal were also found in all beer samples. To the best of our knowledge, an increase in trans-2-butenal during fermentation has not yet been published. Vesely et al. [25] observed an increase during fermentation for another unsaturated aldehyde, trans-2-nonenal, while the concentration of the other aldehydes monitored decreased. The authors suggest that this process is caused by the release of trans-2-nonenal from imine bonds. However, an increase in this aldehyde has also been observed during beer refermentation [14]. As the release from imine bonds may be due to a pH shift, other mechanisms may also be responsible.

Various off-flavors have been attributed to individual carbonyls, most of which have a threshold higher than that of fresh beer [14]; due to synergistic effects, many stale-flavor compounds act as aroma compounds even at low concentrations [11]. In addition, a significant proportion of aldehydes may be bound in complexes, and currently Acácio et al. [26] report 70% bound carbonyls in craft beers. Wort produced by the pressurized wort boiling system with higher aldehyde levels may result in less sensory stable beers.

### 3.5. Sensory Evaluation of Wort

The sensory thresholds (THs) of the studied aldehydes in beer are one to two orders of magnitude higher than in water due to interaction with other sensory-active substances in beer [4]. Also, the THs in beer reported by different authors differ significantly (ASBC). The Strecker aldehydes 2MP, 2MB and 3MB are mainly associated with malty, grainy, various fruity and green flavors. Aldehydes BEN and T2B are associated with bitter almond, PAA with floral, sweet and honey flavors. For HEX, HEP and OCT, bitter and other flavors are reported, T2O and T2N are characterized by stale and carboard flavors, and ACA is associated with grassy, green, fruity and sweet flavors [27]. Concentrations of 2MP, 2MB, 3MB, PAA and T2N in wort were comparable to or higher than the lowest reported TH in beer (see Table S2).

No significant differences were found between the tested variants for any of the evaluated descriptors (see Table S3). This may be due to the low level of carbonyls in the wort, as well as to the overlap of their perception (grainy, malty, sweet) with the unfermented extract or with the bitter substances (bitter) and essential oils (green, floral) from the hops. Sensory analysis of wort is not common, but we attempted to evaluate the differences between the experimental variants in this way. Sensory analysis of beer showed the same result (results not shown). The level of free carbonyls in the fresh beer, except for ACA, did not exceed the lowest reported THs.

## 4. Conclusions

This study showed that wort boiling in the wort-production section has a non-negligible influence on the carbonyl content of this intermediate product of beer production. It was found that the aldehydes form groups that can be expected to behave similarly during wort boiling. During atmospheric wort boiling, the concentration of Strecker aldehydes and some lipid oxidation products decreases. In contrast, the concentration of most carbonyls increases significantly during the process in time- and energy-saving pressurized wort boiling systems. A critical point affecting aldehyde levels in wort is the clarification of wort in the whirlpool, where concentrations of all aldehydes increase due to reduced evaporation. An important finding may be that in cold wort, especially in pressurized systems, the concentration of most aldehydes was significantly higher than in sweet wort. The fermentation process largely eliminated the differences in free carbonyl content in the wort produced by the different boiling systems, but the pressurized wort boiling system could result in lower sensory stability of the beer due to the release of carbonyls from inactive complexes formed during fermentation. This aspect needs further investigation. The results of the presented experiments can be used as a guide for further research or when scaling up to industrial practice.

## Figures and Tables

**Table 1:** Description of wort boiling variants.

Brew	Hop Dosage	Boiling Time (min)	Extract (% *w*/*w*)
ATM1	537 g PE (α-acids 3.35%) at the onset	90	11
ATM2	672 g PE at the onset	90	15
ATM3	645 g PE at the onset	60	11
ATM4	36.5 g EX at the onset	90	11
ATM5	18.2 g EX at the onset, 161 g PE after 30 min, 108 g PE after 70 min	90	11
PWB	537 g PE at the onset	70	11
PDWB	537 g PE at the onset	70	11

PE: hop pellets; EX: hop CO_2_ extract; ATM: atmospheric boil; PWB: pressurized boil; PDWB: dynamic pressurized boil.

**Table 2:** Thiobarbituric acid index (TBA) of wort samples.

Brew	SW	W30	W60	HW	CW
ATM1	34.6 ± 0.4	39.3 ± 1.4	45.0 ± 0.8	47.7 ± 0.2	48.3 ± 0.6
ATM2	33.7 ± 0.2	42.4 ± 0.5	43.8 ± 0.6	47.6 ± 0.6	48.5 ± 0.3
ATM3	27.9 ± 0.4	29.8 ± 0.7	-	33.9 ± 0.7	37.8 ± 0.2
ATM4	31.6 ± 1.0	37.7 ± 0.4	43.6 ± 0.9	46.3 ± 0.8	51.6 ± 0.7
ATM5	31.3 ± 0.7	36.0 ± 0.7	40.7 ± 0.5	44.5 ± 1.0	48.9 ± 0.9
PWB	33.5 ± 0.6	38.3 ± 0.4	42.1 ± 0.6	45.2 ± 0.4	60.5 ± 1.0
PDWB	33.4 ± 0.9	41.0 ± 0.7	46.0 ± 0.9	48.8 ± 0.6	53.1 ± 0.6

SW: sweet wort; W30: after 30 min of wort boiling; W60: after 60 min of wort boiling; HW: hot wort; CW: cold wort.

## Data Availability

The data presented in this study are available in Supplementary Materials here.

## Supplementary Materials

The following supporting information can be downloaded at: https://www.mdpi.com/article/10.3390/foods12163111/s1, Table S1: Extract of wort samples; Table S2: Concentration of carbonyl compounds in wort and beer samples recalculated to original gravity 11% (*w*/*w*); Table S3: Results of the sensory evaluation of the wort.
