# Supplementary material for: Effect of Wort Boiling System and Hopping Regime on Wort and Beer Stale-Flavor Aldehydes
Source: Foods. 2023 Aug 18;12(16):3111. doi: 10.3390/foods12163111 (PMC10453677; doi:10.3390/foods12163111)
Supplement: Supplementary file 1 [file foods-12-03111-s001.zip › foods-2507197-supplementary.pdf]

Supplementary material

Table S1. Extract of wort samples (% w/w).

	SW		W30		W60		HW		CW	
	**R**	**SD**	**R**	**SD**	**R**	**SD**	**R**	**SD**	**R**	**SD**
ATM1	10.75	0.03	11.20	0.02	11.48	0.03	11.76	0.06	11.62	0.02
ATM2	15.03	0.08	15.64	0.06	15.90	0.15	16.44	0.04	15.87	0.10
ATM3	11.10	0.30	11.41	0.05	-	-	11.65	0.07	11.36	0.10
ATM4	10.71	0.15	11.08	0.08	11.35	0.10	11.63	0.05	11.70	0.06
ATM5	10.96	0.16	11.28	0.08	11.56	0.06	11.86	0.06	11.46	0.06
PWB	10.85	0.11	10.92	0.07	10.93	0.03	10.93	0.03	10.92	0.02
PDWB	10.83	0.13	10.97	0.07	10.97	0.05	11.04	0.04	10.94	0.06

R: Average; SD: Standard deviation. SW: sweet wort, W30: after 30 min of wort boiling, W60: after 60 min of wort boiling, HW: hot wort, CW: cold wort.

Table S2. Concentration of carbonyl compounds in wort and beer samples (μg/L) recalculated to original gravity 11 % (w/w).

Compound (TH)	Brew	SW	W30	W60	HW	CW	Beer
Acetaldehyde (1114)	ATM1	625[a]	300[a]	255[a]	199[a]	359[ab]	10539[c]
	ATM2	917[c]	657[cd]	347[a]	224[a]	404[ab]	3055[a]
	ATM3	853[bc]	575[bcd]	---	310[b]	435[ab]	10274[c]
	ATM4	809[abc]	369[ab]	280[a]	186[a]	283[a]	9503[c]
	ATM5	704[abc]	472[abc]	313[a]	448[c]	490[b]	12796[d]
	PWB	699[ab]	1361[e]	2166[c]	1514[e]	1201[d]	9873[c]
	PDWB	823[abc]	742[d]	971[b]	642[d]	793[c]	5688[b]
Acetone (200000)	ATM1	603[ab]	485[a]	423[a]	313[a]	291[ab]	106.8[ab]
	ATM2	835[c]	914[cd]	499[a]	272[a]	285[ab]	107.6[ab]
	ATM3	718[abc]	903[bcd]	---	514[b]	422[bc]	141.7[bc]
	ATM4	788[bc]	511[ab]	513[a]	329[a]	206[a]	77.1[a]
	ATM5	559[a]	544[abc]	453[a]	724[c]	555[c]	156.4[c]
	PWB	603[ab]	2108[e]	2242[c]	2302[e]	1314[e]	412.2[d]
	PDWB	780[bc]	981[d]	1246[b]	902[d]	853[d]	177.7[c]
2-Methylpropanal (50)	ATM1	83.1[ab]	38.1[a]	41.2[a]	40.0[a]	88.8[a]	14.65[c]
	ATM2	115.2[d]	79.4[b]	68.3[b]	53.3[b]	119.4[bc]	13.63[bc]
	ATM3	89.5[abc]	55.3[a]	---	42.7[a]	87.3[a]	9.34[a]
	ATM4	92.0[bc]	41.2[a]	44.2[a]	42.2[a]	86.4[a]	13.45[bc]
	ATM5	69.5[a]	40.5[a]	37.9[a]	62.0[b]	97.1[ab]	14.65[c]
	PWB	75.1[ab]	157.4[c]	192.0[d]	143.5[d]	155.5[d]	11.60[ab]
	PDWB	108.7[cd]	80.6[b]	120.5[c]	82.2[c]	133.0[cd]	9.51[a]
3-Methylbutan-2-one (5)	ATM1	0.32[ab]	2.02[bc]	1.30[a]	0.89[a]	3.75[b]	0.56[b]
	ATM2	0.29[a]	2.28[c]	1.34[a]	0.78[a]	3.69[b]	0.61[b]
	ATM3	0.72[c]	3.55[d]	---	1.95[b]	5.69[c]	0.99[c]
	ATM4	0.39[ab]	1.12[ab]	1.01[a]	0.65[a]	1.49[a]	0.21[a]
	ATM5	0.44[b]	0.75[a]	1.15[a]	2.59[c]	3.10[b]	0.51[b]
	PWB	0.67[c]	5.62[e]	7.50[c]	6.00[e]	6.46[c]	1.09[c]
	PDWB	1.18[d]	2.57[cd]	5.90[b]	4.99[d]	5.94[c]	0.72[b]
2-Methylbutanal (45)	ATM1	40.7[ab]	22.5[a]	27.5[a]	26.0[a]	56.3[a]	5.11[bcd]
	ATM2	53.4[c]	43.7[b]	46.1[b]	38.2[bc]	82.6[b]	3.80[a]

	ATM3	43.1[ab]	28.7[a]	---	24.5[a]	51.9[a]	4.52[abc]
	ATM4	47.2[bc]	24.5[a]	30.5[a]	30.6[ab]	59.1[a]	5.59[d]
	ATM5	36.9[a]	23.2[a]	24.3[a]	39.5[c]	64.7[a]	5.90[d]
	PWB	39.0[a]	76.1[c]	108.0[d]	74.3[e]	88.4[b]	5.32[cd]
	PDWB	52.6[c]	40.3[b]	68.5[c]	50.7[d]	82.6[b]	4.13[ab]
3-Methylbutanal (56)	ATM1	83.7[ab]	47.1[a]	52.7[a]	55.6[a]	140.4[ab]	16.21[bcd]
	ATM2	113.3[c]	91.7[b]	93.1[b]	75.0[b]	194.7[c]	12.31[a]
	ATM3	81.7[ab]	55.6[a]	---	52.9[a]	117.0[a]	13.99[abc]
	ATM4	97.0[bc]	49.4[a]	57.8[a]	62.3[ab]	139.2[ab]	15.91[bcd]
	ATM5	77.4[a]	53.9[a]	61.4[a]	99.9[c]	178.1[bc]	18.18[d]
	PWB	79.9[a]	178.9[c]	277.0[d]	162.8[e]	239.9[d]	16.67[cd]
	PDWB	103.0[c]	91.3[b]	154.9[c]	124.9[d]	209.6[cd]	13.44[ab]
trans-2-Butenal (8000)	ATM1	0.53[a]	0.59[a]	0.66[a]	0.67[ab]	0.80[a]	1.01[a]
	ATM2	0.58[a]	0.81[a]	0.62[a]	0.50[a]	0.62[a]	0.97[a]
	ATM3	0.58[a]	0.81[a]	---	0.82[bc]	0.72[a]	1.51[c]
	ATM4	0.52[a]	0.60[a]	0.62[a]	0.52[a]	0.63[a]	1.03[ab]
	ATM5	0.47[a]	0.63[a]	0.59[a]	0.86[bc]	0.74[a]	1.28[bc]
	PWB	0.54[a]	0.82[a]	0.96[b]	0.96[c]	0.76[a]	1.10[ab]
	PDWB	0.59[a]	0.64[a]	0.78[ab]	0.80[bc]	0.81[a]	1.11[ab]
Hexanal (88)	ATM1	13.64[bc]	12.58[a]	11.17[a]	8.75[b]	10.60[ab]	2.03[ab]
	ATM2	10.33[a]	10.91[a]	7.66[a]	4.28[a]	9.68[ab]	1.56[ab]
	ATM3	15.93[d]	15.02[a]	---	12.84[cd]	10.22[ab]	1.56[ab]
	ATM4	14.31[cd]	10.76[a]	16.43[b]	9.59[bc]	7.93[a]	1.46[ab]
	ATM5	13.88[bc]	13.25[a]	10.43[a]	13.53[d]	12.15[bc]	2.88[c]
	PWB	12.39[b]	34.08[b]	35.86[c]	24.51[f]	21.59[d]	2.17[bc]
	PDWB	13.53[bc]	15.31[a]	20.83[b]	18.45[e]	14.52[c]	1.25[a]
Heptanal (80)	ATM1	1.18[d]	1.05[c]	1.18[b]	0.75[b]	1.99[c]	0.39[ab]
	ATM2	0.63[a]	0.66[a]	0.64[a]	0.44[a]	1.52[ab]	0.28[a]
	ATM3	1.00[cd]	0.99[bc]	---	0.96[bc]	1.79[bc]	0.29[a]
	ATM4	0.90[bc]	1.02[bc]	0.91[ab]	0.82[bc]	1.32[a]	0.39[ab]
	ATM5	0.73[ab]	0.74[ab]	0.58[a]	0.82[bc]	1.27[a]	0.43[b]
	PWB	0.55[a]	1.23[cd]	2.54[d]	1.06[c]	1.82[bc]	0.38[ab]
	PDWB	0.98[bcd]	1.46[d]	1.95[c]	2.05[d]	2.37[d]	0.32[ab]

	ATM1	2.47^{b}	2.77^{d}	3.47^{ab}	1.46^{ab}	3.91^{d}	2.44^{bc}
	ATM2	0.32^{a}	1.48^{abc}	1.88^{ab}	0.50^{a}	1.44^{a}	1.42^{a}
	ATM3	1.38^{ab}	1.78^{abcd}	---	1.79^{ab}	3.20^{c}	1.63^{ab}
Octanal (40)	ATM4	0.80^{ab}	2.53^{cd}	2.63^{ab}	2.47^{bc}	2.22^{b}	2.00^{abc}
	ATM5	0.65^{ab}	0.78^{a}	0.95^{a}	1.20^{ab}	1.15^{a}	2.51^{c}
	PWB	0.63^{ab}	1.07^{ab}	2.21^{ab}	1.05^{ab}	1.50^{a}	2.26^{abc}
	PDWB	0.82^{ab}	2.10^{bcd}	4.60^{b}	3.29^{c}	3.03^{c}	1.46^{a}
	ATM1	29.0^{b}	32.6^{bc}	42.5^{ab}	43.1^{b}	76.6^{bc}	5.27^{ab}
	ATM2	29.9^{b}	41.4^{cd}	45.7^{ab}	40.8^{b}	68.3^{b}	5.08^{ab}
	ATM3	15.4^{a}	20.3^{a}	---	17.2^{a}	36.3^{a}	5.61^{b}
Furfural (15000)	ATM4	25.7^{b}	44.9^{d}	54.6^{ab}	46.7^{b}	87.6^{cd}	4.67^{ab}
	ATM5	16.4^{a}	28.1^{ab}	36.0^{a}	48.7^{bc}	62.3^{b}	4.13^{a}
	PWB	18.0^{a}	32.1^{b}	58.4^{b}	74.8^{d}	68.7^{b}	5.34^{ab}
	PDWB	24.8^{b}	35.5^{bc}	82.8^{c}	59.8^{c}	103.2^{d}	4.89^{ab}
	ATM1	0.03^{a}	0.10^{a}	0.08^{a}	0.05^{a}	0.07^{a}	0.02^{a}
	ATM2	0.03^{a}	0.03^{a}	0.03^{a}	0.02^{a}	0.05^{a}	0.02^{a}
	ATM3	0.05^{a}	0.05^{a}	---	0.06^{a}	0.07^{a}	0.03^{a}
trans-2-Octenal 0.20)	ATM4	0.03^{a}	0.08^{a}	0.16^{a}	0.10^{a}	0.05^{a}	0.02^{a}
	ATM5	0.08^{a}	0.08^{a}	0.06^{a}	0.09^{a}	0.05^{a}	0.03^{a}
	PWB	0.09^{a}	0.13^{a}	0.14^{a}	0.13^{a}	0.06^{a}	0.01^{a}
	PDWB	0.05^{a}	0.08^{a}	0.10^{a}	0.14^{a}	0.11^{b}	0.02^{a}
	ATM1	0.10^{b}	0.06^{a}	0.06^{a}	0.06^{a}	0.07^{ab}	0.01^{a}
	ATM2	0.03^{a}	0.06^{a}	0.04^{a}	0.03^{a}	0.05^{ab}	0.02^{a}
	ATM3	0.05^{ab}	0.06^{a}	---	0.04^{a}	0.04^{ab}	0.01^{a}
trans-2-Nonenal (0,03)	ATM4	0.05^{ab}	0.05^{a}	0.06^{a}	0.04^{a}	0.05^{ab}	0.01^{a}
	ATM5	0.03^{a}	0.02^{a}	0.04^{a}	0.06^{a}	0.04^{ab}	0.02^{a}
	PWB	0.03^{a}	0.03^{a}	0.04^{a}	0.04^{a}	0.03^{a}	0.02^{a}
	PDWB	0.06^{ab}	0.05^{a}	0.05^{a}	0.08^{a}	0.09^{b}	0.02^{a}
	ATM1	7.50^{bc}	5.27^{ab}	4.45^{ab}	3.66^{a}	3.80^{a}	3.06^{a}
	ATM2	6.04^{ab}	4.37^{a}	3.77^{a}	2.92^{a}	3.43^{a}	2.94^{a}
Benzaldehyde (515)	ATM3	5.71^{a}	4.82^{ab}	---	3.59^{a}	3.79^{a}	3.74^{a}
	ATM4	7.69^{c}	4.99^{ab}	4.30^{ab}	3.51^{a}	3.36^{a}	3.94^{a}
	ATM5	9.95^{e}	6.08^{ab}	6.06^{b}	6.85^{b}	6.44^{b}	3.04^{a}

	PWB	9.34[de]	11.66[c]	13.06[d]	11.70[c]	11.22[c]	3.62[a]
	PDWB	8.09[cd]	7.13[b]	8.50[c]	6.71[b]	7.16[b]	3.47[a]
Phenylacetaldehyde (18)	ATM1	118.0[bc]	107.8[ab]	114.8[a]	99.6[b]	122.9[b]	14.53[ab]
	ATM2	108.1[ab]	120.6[b]	130.6[ab]	116.3[bc]	151.9[bc]	16.37[bc]
	ATM3	104.0[ab]	92.3[a]	---	80.3[a]	86.9[a]	11.65[a]
	ATM4	133.8[c]	124.3[b]	122.5[ab]	119.2[c]	163.9[cd]	17.14[bc]
	ATM5	120.9[bc]	121.3[b]	145.5[bc]	175.1[d]	169.8[cd]	20.17[c]
	PWB	92.4[a]	162.9[c]	196.3[d]	166.4[d]	194.2[d]	17.17[bc]
	PDWB	111.4[abc]	130.2[b]	162.9[c]	134.2[c]	176.9[cd]	15.66[b]

Lower case letters indicate significant differences at the a=0.05 level between wort boiling variants for a compound in a given column, e.g. SW.
TH: lowest reported sensory threshold in beer [27].

Table S3. Results of the sensory evaluation of the wort.

Descriptor/Brew	ATM1	ATM2	ATM3	ATM4	ATM5	PWB	PDWB
palate fullness	2.9 ^{a}	2.9 ^{a}	2.7 ^{a}	2.9 ^{a}	2.8 ^{a}	2.4 ^{a}	2.9 ^{a}
bitterness	4.0 ^{a}	3.9 ^{a}	3.5 ^{a}	3.5 ^{a}	4.0 ^{a}	3.7 ^{a}	3.9 ^{a}
bitterness character	3.3 ^{a}	3.1 ^{a}	3.1 ^{a}	3.2 ^{a}	3.3 ^{a}	3.6 ^{a}	3.3 ^{a}
sweetness	2.2 ^{a}	2.4 ^{a}	2.5 ^{a}	3.1 ^{a}	2.5 ^{a}	2.3 ^{a}	2.3 ^{a}
astringency	1.8	1.6 ^{a}	1.4 ^{a}	1.8 ^{a}	2.3 ^{a}	1.8 ^{a}	1.8 ^{a}
sourness	0.8 ^{a}	1.0 ^{a}	1.1 ^{a}	0.8 ^{a}	0.8 ^{a}	1.1 ^{a}	0.5 ^{a}
grainy	1.2 ^{a}	1.1 ^{a}	0.9 ^{a}	1.3 ^{a}	1.5 ^{a}	0.7 ^{a}	1.8 ^{a}
malty	1.2 ^{a}	1.3 ^{a}	1.7 ^{a}	1.8 ^{a}	1.7 ^{a}	1.5 ^{a}	1.4 ^{a}
biscuity	*	*	0.9 ^{a}	0.9 ^{a}	*	0.6 ^{a}	0.9 ^{a}
nutty	0.7 ^{a}	*	0.6 ^{a}	1.0 ^{a}	1.0 ^{a}	*	1.1 ^{a}
honey-like	1.0 ^{a}	1.0 ^{a}	0.9 ^{a}	1.0 ^{a}	0.7 ^{a}	1.0 ^{a}	0.9 ^{a}
papery	NIF	NIF	*	NIF	NIF	NIF	NIF
musty	NIF	NIF	NIF	NIF	NIF	NIF	*
grassy	1.5 ^{a}	0.7 ^{a}	1.2 ^{a}	0.9 ^{a}	*	1.1 ^{a}	*
caramel	*	*	*	*	*	*	*
bitter caramel	0.7 ^{a}	*	*	1.2 ^{a}	1.4 ^{a}	1.1 ^{a}	*
bready	NIF	*	*	1.1	*	*	*
medicinal	NIF	NIF	NIF	NIF	NIF	NIF	NIF
candyfloss	NIF	*	*	*	NIF	NIF	NIF
mouse	NIF	NIF	NIF	NIF	NIF	NIF	NIF
chemical	NIF	NIF	NIF	NIF	NIF	NIF	NIF
burnt	1.6 ^{a}	1.1 ^{a}	1.2 ^{a}	1.0 ^{a}	1.2 ^{a}	*	*
roasted	*	*	*	*	*	*	1.1
floral	1.4 ^{a}	*	1.0 ^{a}	0.7 ^{a}	*	0.9 ^{a}	0.9 ^{a}
fruity	*	*	*	*	*	*	*
green grass	*	*	*	*	*	*	0.7
herbal	1.4 ^{a}	*	1.2 ^{a}	1.1 ^{a}	*	1.0 ^{a}	0.9 ^{a}
spicy	1.5 ^{a}	*	1.0 ^{a}	1.3 ^{a}	*	1.6 ^{a}	0.9 ^{a}
woody	*	*	*	1.2	*	*	*

NIF: Not identified by any evaluator; * Identified by less than a third of evaluators. Lower case letters indicate significant differences at the a=0.05 level between wort boiling variants.
